# Mitochondrial Bioenergetic Alterations in Mouse Neuroblastoma Cells Infected with Sindbis Virus: Implications to Viral Replication and Neuronal Death

**DOI:** 10.1371/journal.pone.0033871

**Published:** 2012-04-02

**Authors:** Leandro Silva da Costa, Ana Paula Pereira da Silva, Andrea T. Da Poian, Tatiana El-Bacha

**Affiliations:** 1 Instituto de Bioquímica Médica, Universidade Federal do Rio de Janeiro, Cidade Universitária, Rio de Janeiro, Rio de Janeiro, Brazil; 2 Setor de Bioquímica, Departamento de Química, Instituto de Ciências Exatas, Universidade Federal Rural do Rio de Janeiro, Seropédica, Rio de Janeiro, Brazil; Université Joseph Fourier, France

## Abstract

The metabolic resources crucial for viral replication are provided by the host. Details of the mechanisms by which viruses interact with host metabolism, altering and recruiting high free-energy molecules for their own replication, remain unknown. Sindbis virus, the prototype of and most widespread alphavirus, causes outbreaks of arthritis in humans and serves as a model for the study of the pathogenesis of neurological diseases induced by alphaviruses in mice. In this work, respirometric analysis was used to evaluate the effects of Sindbis virus infection on mitochondrial bioenergetics of a mouse neuroblastoma cell lineage, Neuro 2a. The modulation of mitochondrial functions affected cellular ATP content and this was synchronous with Sindbis virus replication cycle and cell death. At 15 h, irrespective of effects on cell viability, viral replication induced a decrease in oxygen consumption uncoupled to ATP synthesis and a 36% decrease in maximum uncoupled respiration, which led to an increase of 30% in the fraction of oxygen consumption used for ATP synthesis. Decreased proton leak associated to complex I respiration contributed to the apparent improvement of mitochondrial function. Cellular ATP content was not affected by infection. After 24 h, mitochondria dysfunction was clearly observed as maximum uncoupled respiration reduced 65%, along with a decrease in the fraction of oxygen consumption used for ATP synthesis. Suppressed respiration driven by complexes I- and II-related substrates seemed to play a role in mitochondrial dysfunction. Despite the increase in glucose uptake and glycolytic flux, these changes were followed by a 30% decrease in ATP content and neuronal death. Taken together, mitochondrial bioenergetics is modulated during Sindbis virus infection in such a way as to favor ATP synthesis required to support active viral replication. These early changes in metabolism of Neuro 2a cells may form the molecular basis of neuronal dysfunction and Sindbis virus-induced encephalitis.

## Introduction

A central axis of the host response to virus infection is the modulation of pathways involved in cell survival and death. In this respect, several responses are developed by host cells that may control virus replication and infection. On the other hand, viruses have developed strategies to counteract host responses. In different hosts and viruses, many of these responses involve alterations in cellular metabolism [1]. It has been shown that an increase in the uptake and utilization of glucose are events observed during the infection of mammalian cells with alpha, rhabdo, herpes, ortomyxo and retroviruses [2], [3], [4], [5], [6], [7]. The increased utilization of glucose by infected cells supplies both ATP and biosynthetic precursors required for virus replication, as observed in Mayaro and Sindbis viruses infections [2], [3], [4]. However, this may represent an anti-viral cellular response, as in the case of Human Immunodeficiency Virus (HIV) infection [8]. Additionally, the increase in glucose uptake observed in HIV infection distinguished an acute from a chronic infection phenotype [9]. Likewise, the up regulation of lipid biosynthesis has been demonstrated to be important for the replication of human Cytomegalovirus (CMV), Influenza, Hepatitis B and C viruses [8], [10], [11], suggesting that lipid metabolism-related pathways might be targets for anti-viral therapy.

Mitochondria are also implicated directly and indirectly in several host and viral responses. These organelles participate in major early anti-viral immune responses through mitochondria-associated adapters molecules, such as MAVS [12]. Additionally, viral proteins inserted in mitochondrial membranes present either anti- and/or pro-apoptotic functions, affecting survival/death pathways. In this respect, phenomena directly associated to virus-induced apoptosis that appear to occur with different types of cellular infections reflect alterations in mitochondrial membranes permeability and dissipation of mitochondrial membrane potential (ΔΨ_M_) [13], [14], [15], [16]. Viral infections may also interfere with mitochondrial bioenergetics by means of effects on cellular respiratory functions and oxidative pathways, which were shown to be important for viral replication and consequently should represent early responses to viral infection. In this regard, it has been shown that fibroblasts infected with human CMV displayed, in addition to increased flux through glycolysis and ATP production, presented an increased content of tricarboxylic acid cycle intermediates [5]. Furthermore, results from our group demonstrated that human hepatic cells infected with Dengue virus exhibited an increase in mitochondrial respiration and decrease in ATP content, events which preceded cell death [17].

Sindbis virus (SinV), the prototype and most widespread alphavirus, is a single-stranded positive-sense RNA virus that causes outbreaks of arthritis and rash in Northern Europe and Southern Africa [18]. In mice, SinV is able to infect neurons and serves as a model for the study of the pathogenesis of neurological diseases induced by alphaviruses. Animal age and virulence are determinants for the outcome of infection, and neuronal damage is related to cell death, which was shown to be caused by apoptosis [19], [20]. Neurovirulence is related to the ability of SinV to decrease type I and II IFNs immune responses [21], apparently via the JAK/STAT axis [22], and selective autophagy plays an important role in the protection against lethal infection of the central nervous system [23].

Regarding the involvement of mitochondria in SinV-induced neuronal death, about 20 years ago it was shown that overexpression of Bcl-2 protein in mice neuroblastoma cells converted lytic to persistent infection [24]. Beclin-1 [25] and Bax [26] expression were also shown to prevent neuronal death in newborn mice, and antioxidant treatment also protected mice from apoptotic cell death [27]. These facts clearly demonstrated that modulation of mitochondrial functions played an important role in the outcome of SinV infection in neurons. However, despite the fact that mitochondrial disorders are often observed in neurodegenerative diseases, a detailed characterization of mitochondrial bioenergetics and its relationship, if any, to the replication cycle of SinV in neuroblastoma cells has not yet been adequately addressed.

The present study characterized morphologically and biochemically the infection of mouse neuroblastoma cells Neuro 2a with SinV and evaluated mitochondrial bioenergetics and energy homeostasis during the course of infection. The observed changes in mitochondrial bioenergetics included alterations in respiratory properties, which preceded apoptotic cell death and seemed to be related to an increased efficiency for ATP synthesis. Presumably, these changes were selected to maximize replication and may form the molecular basis for neuronal dysfunction and SinV-induced encephalitis.

## Results

### Characterization of SinV replication on Neuro 2a cells

**Figure 1:**
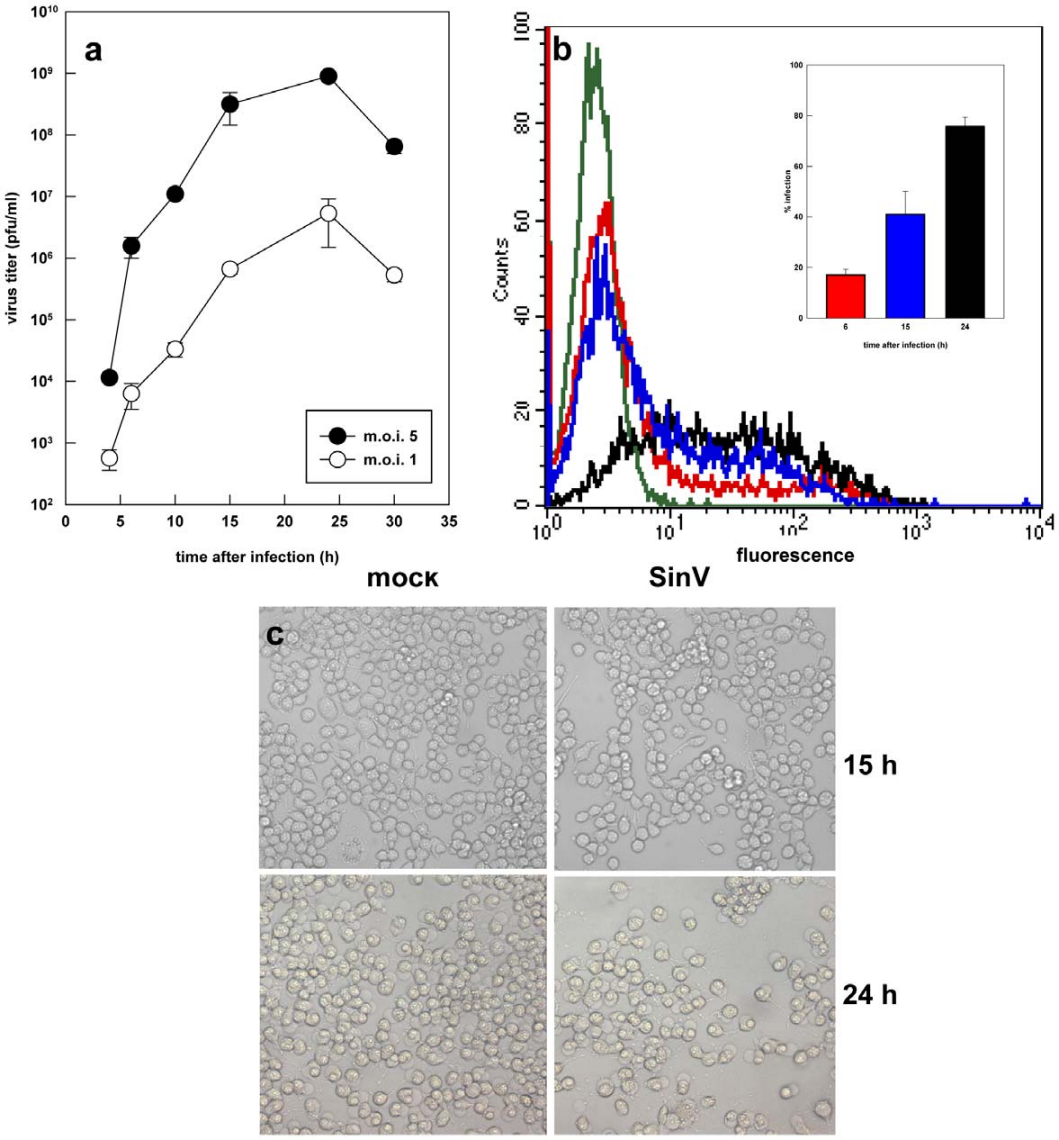
Characterization of SinV infection of Neuro 2a cells. (a) Neuro 2a cells were infected with SinV at MOI 1 (open symbols) and 5 (filled symbols) and after different time points viral titers were quantified by plaque assays. (b) The efficient of infection at MOI 5 was evaluated by flow cytometry, as described in materials and methods. (c) Morphological alterations of Neuro 2a cells infected with SinV: At 24 h of infection, cells were less confluent and with cellular membrane protrusions resembling membrane blebs, which are morphological alterations observed during the apoptotic process. (a,b) Values represent mean ± SEM; n = 4; (c) Representative fields of mock-infected and Neuro 2a infected cells at 15 and 24 h.

The first set of experiments was conducted to characterize the replication of SinV on Neuro 2a cells. Although SinV replication was commonly characterized in N18 cells, another mouse neuroblastoma cell lineage, the selection of Neuro 2a cells relates to the fact that they are also used for the study of encephalitis caused by flaviviruses, such as induced by Dengue, Japanese encephalitis and West Nile viruses [28], [29]. This brings the possibility for a better comprehension of the replication strategies of different viruses and of the molecular basis of viral pathogenesis, possibly contributing to therapy development. Additionally, Neuro 2a cells are good model for studies of oxidative stress and mitochondrial function [30], [31], [32]. Figure 1a shows the replication profile of SinV in Neuro 2a cells exposed to a multiplicity of infection (MOI) of 1 and 5. It can be seen that the production of infectious viral particles began after 6 h and that viral replication peaked around 15 h post-infection, regardless of the MOI utilized. Additionally, the replication curves were similar in the two conditions, although viral yield was 3 orders of magnitude larger with MOI 5 when compared to MOI 1. Thus, MOI 5 was then used in all subsequent experiments.

Further analysis of SinV replication in Neuro 2a cells were carried out by quantifying the efficiency of infection by means of flow cytometry. Figure 1b shows that after 6, 15 and 24 h, the percentage of infected cells were 18, 37 and 68%, respectively. These results indicate that the profile of SinV replication and virus yield in Neuro 2a cells is very similar to that of the cell line N18 [33] and, therefore, that they are also good models for SinV-host interactions studies.

As observed by phase contrast microscopy, only after 24 h of infection Neuro 2a cells exhibited discrete morphological differences in comparison to control cells (Figure 1c). It can be seen that SinV infection resulted in cells that were less confluent displaying cell membrane protrusions resembling membrane blebs, which is one of the morphological alterations observed during the apoptotic process.

### SinV infection affects Neuro 2a cells viability and induces apoptotic cell death

**Figure 2:**
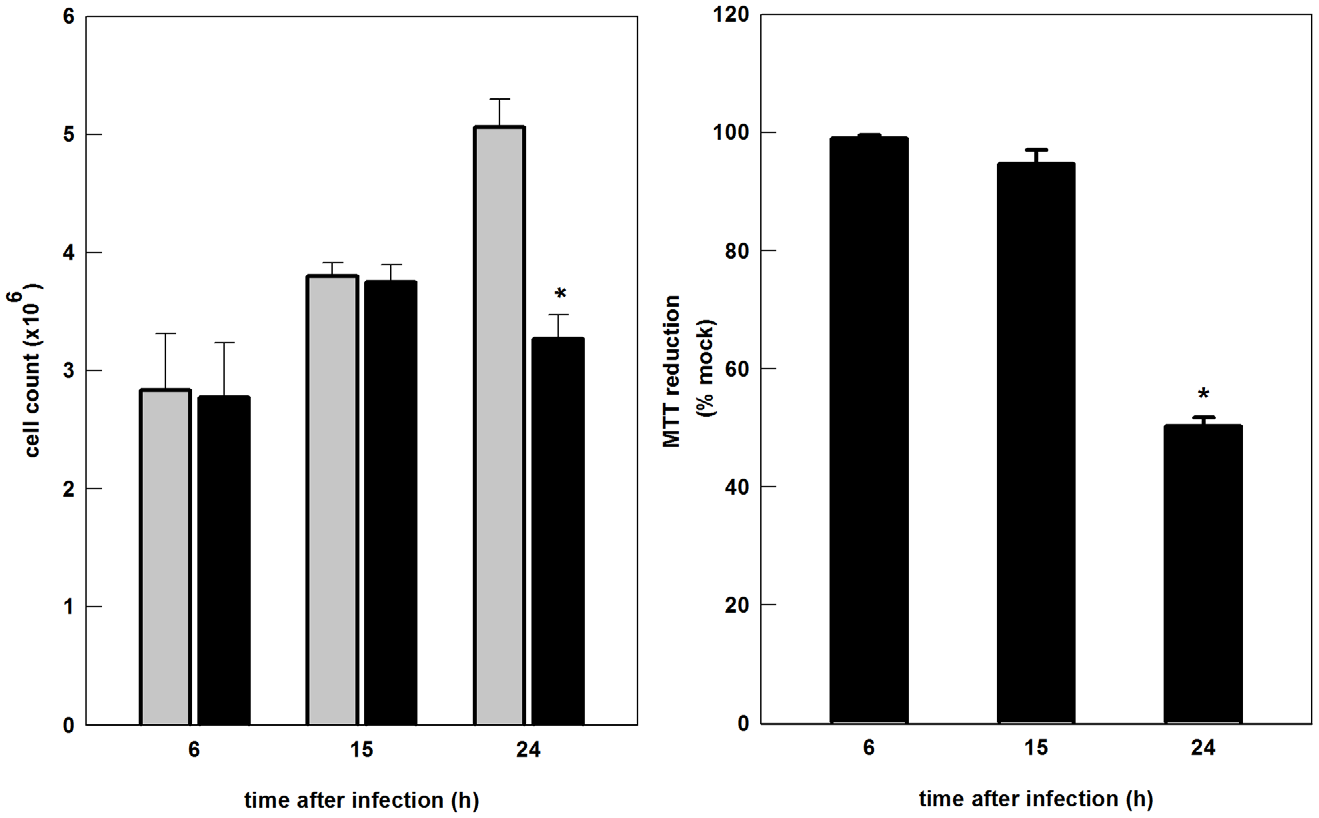
SinV infection causes a decrease in Neuro 2a cells number and viability. Neuro 2a cells were infected with SinV at MOI 5 and after 6, 15 and 24 h (a) total cell count was performed with trypan blue dye exclusion assay and (b) cell viability was evaluated by the MTT method. Mock-infected (gray bars) and SinV-infected (black bars) Neuro 2a cells. Values represent mean ± SEM; N = 4, *P<0.05 compared to mock-infected cells.

**Figure 3:**
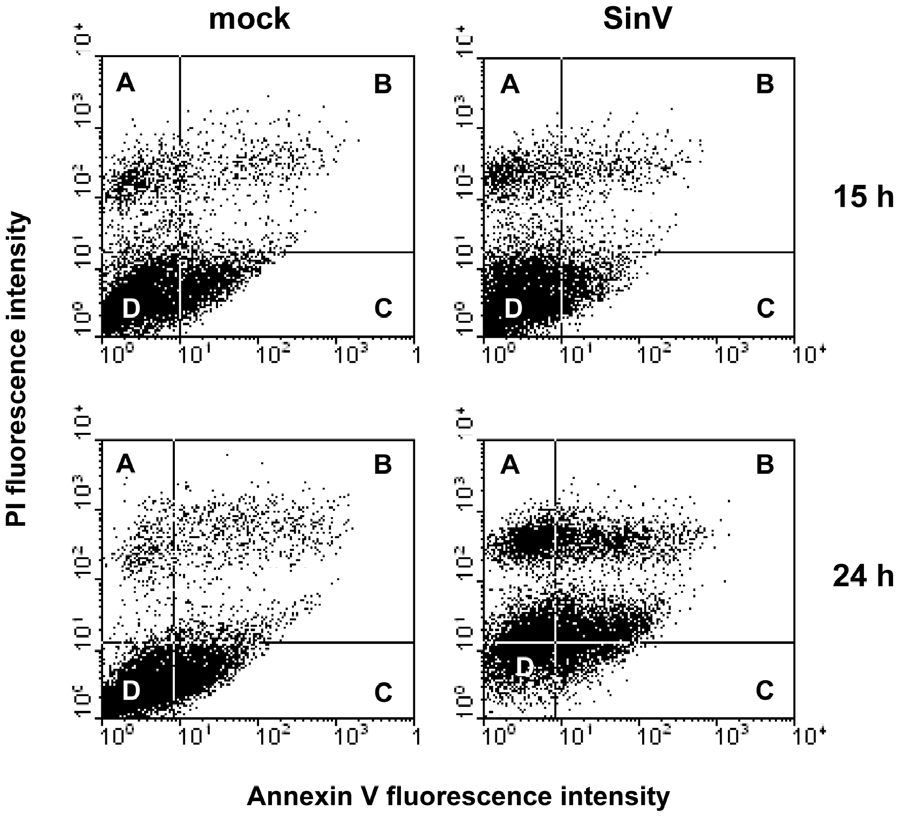
SinV induces apoptotic and necrotic Neuro 2a cells death. Neuro2a cells were stained with annexin V-FITC/PI and analyzed by flow cytometry. Letters represent the proportion of: A: Dead cells (Annexin V-FITC−/PI+); B: Late apoptotic/necrotic cells (Annexin V-FITC+/PI+); and C: Early apoptotic cells (Annexin V-FITC+/PI−); and D: Viable cells.

In an attempt to characterize the effects of SinV infection on Neuro 2a viability, we performed experiments using MTT and trypan blue dye exclusion assays. The results showed that SinV promoted a decrease of two orders of magnitude in cell number (Figure 2a) and 45% in cell viability (Figure 2b) after 24 h infection. No differences were observed after 6 and 15 h after infection. It is important to mention that the percentage of viable cells remained constant in the mock-infected group at all time points studied. Since it is well known that neuronal infection with SinV results in cell death, the mechanisms of Neuro 2a death were characterized by double labeling cells with Annexin V-FITC and Propidium Iodide (PI) and evaluated by means of flow cytometry. The results in Figure 3 and Table 1 show that the amount of early apoptotic cells did not differ from 15 to 24 h post-infection. On the other hand, the percentage of late apoptotic/necrotic cells increased 5 fold during the course of SinV infection, where in 15 h it accounted for approximately 7% and after 24 h, 37%. Additionally, dead cells corresponded to 13% and 28% after 15 and 24 h after SinV infection, respectively. Comparing the results obtained from mock-infected labeled cells with those after 15 h of SinV infection, it can be seen that the percentage of viable and apoptotic/necrotic cells did not differ significantly between these two groups. Taken into account these results, major changes occurred on cell viability between 15 and 24 h after SinV infection, despite the fact that viral titers are precisely the same in these two conditions, as shown in Figure 1a. Therefore, the set of experiments described next were performed to evaluate possible alterations on mitochondrial function and energy homeostasis following 15 and 24 h of SinV infection of Neuro 2a cells.

**Table 1:** Proportion of Annexin V-FITC/PI labeled Neuro 2a cells after 15 and 24 h of SinV infection.

Time (h)	Dead(%±SE)		Late apoptotic/necrotic(%±SE)		Early apoptotic(%±SE)		Viable(%±SE)	
	(annexin V-FITC−/PI+)		(annexin V-FITC+/PI+)		(annexin V-FITC+/PI−)		(annexin V-FITC−/PI−)	
	Mock	SinV	Mock	SinV	Mock	SinV	Mock	SinV
15	14.1±5.3	13.1±2.5	7.4±1.3	6.7±1.3	12.3±4.3	9.0±1.4	67.3±1.3	67.6±6.0
24	9.5±2.4	28.4±4.6	11.1±1.1	37.4±2.0	6.2±0.9	9.5±1.7	69.4±2.4	24.8±6.8

### SinV infection affects mitochondrial function in Neuro 2a cells

**Figure 4:**
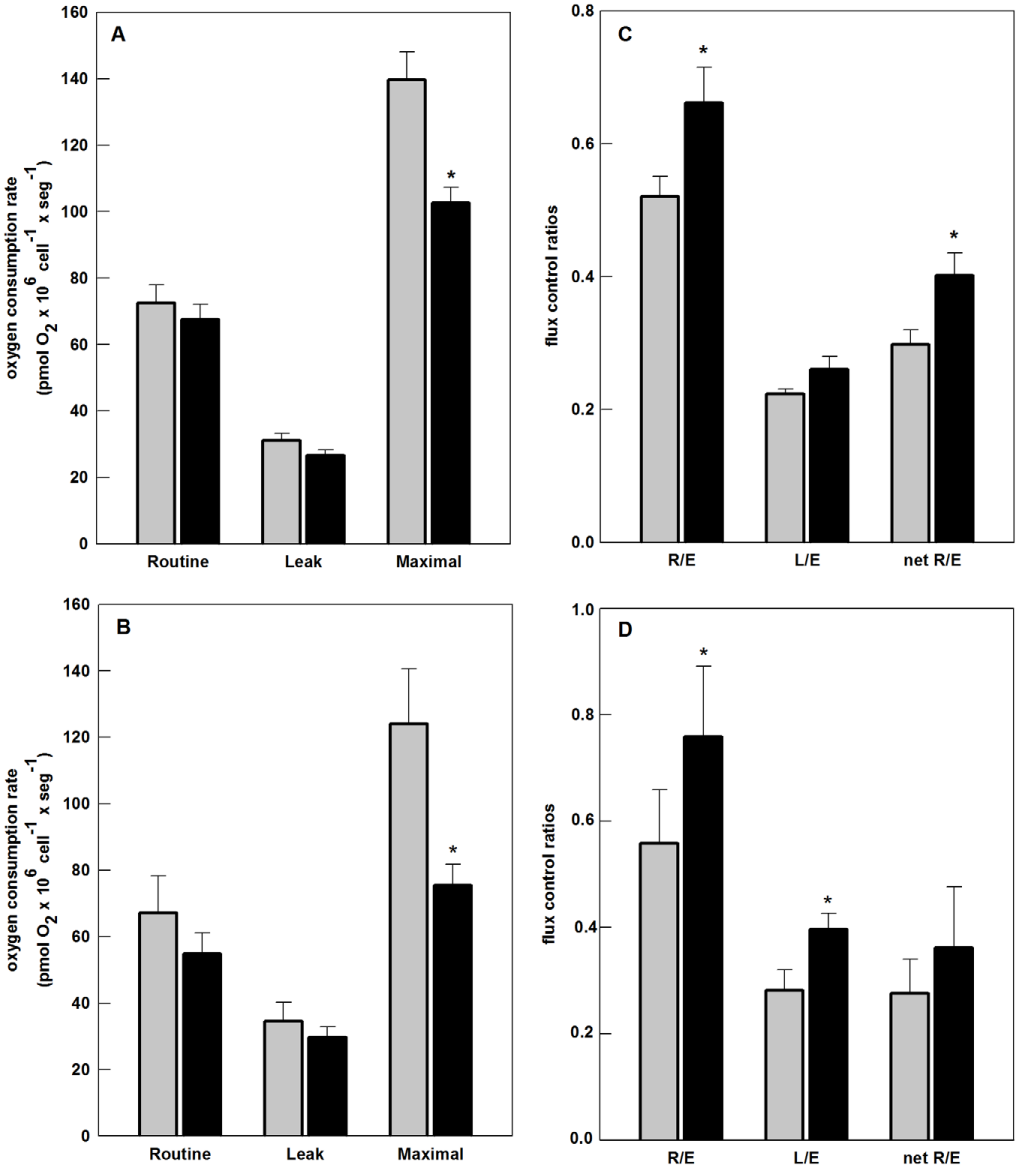
High-resolution respirometry reveals mitochondrial dysfunction in intact Neuro 2a cells infected with SinV. Oxygen consumption rates were measured on intact Neuro 2a cells in DMEM with 5 mM glucose without SFB. Routine (R) oxygen consumption rates in the coupled state; Leak (L) oxygen consumption rates after addition of oligomycin, i.e., not coupled to ATP synthesis; Maximal (E) oxygen consumption rates after FCCP addition, which reflects ETS capacity. ETS capacity was used to normalize and to calculate respiratory flux control ratios R/E, L/E and netR/E. Mock-infected (gray bars) and SinV-infected (black bars) after 15 h (a,b) and 24 h (c,d). Values represent mean ± SEM; N = 5, ^#^ *P*<0.07 compared to mock-infected cells; *P<0.05 compared to mock-infected cells.

Oxygen consumption was evaluated in intact and permeabilized Neuro 2a cells, using high-resolution respirometry. Figure 4a shows the respiratory parameters of intact Neuro 2a cells after 15 h of SinV infection. It can be seen that Routine respiration of Neuro 2a cells (in culture medium containing 5 mM glucose) did not differ between mock-infected and infected cells and the average rates were, respectively, 72.6 and 67.5 pmoles O_2_×10^6^ cells^−1^×seg^−1^. Oxygen consumption rate in the presence of oligomycin, a compound that inhibits ATP synthase activity, is referred to as Leak respiration since it evaluates oxygen consumption uncoupled to ATP synthesis. Leak respiration of mock-infected Neuro 2a cells was 31.1 and that of SinV-infected cells was 26.6 pmoles O_2_×10^6^ cells^−1^×seg^−1^. Although not significantly different, there was a statistical tendency (*P*<0.07) for Leak respiration to be decreased after 15 h of SinV infection. Additionally, the results in Figure 4a show that electron transport system (ETS) capacity [oxygen consumption rate induced by carbonyl cyanide p-(trifluoromethoxy) phenylhydrazone (FCCP)] was reduced in 36% in Neuro 2a cells after 15 h of SinV infection, whereas the average rates in mock-infected and SinV-infected Neuro 2a cells were 139.6 and 102.7 pmoles O_2_×10^6^ cells^−1^×seg^1^, respectively.

The results in Figure 4a were used to calculate the respiratory flux control ratios presented in Figure 4b. The Routine flux control ratio (R/E), which expresses how close Routine respiration works from maximum ETS capacity, was significantly higher in infected cells (0.66) when compared to mock-infected cells (0.52), indicating that after 15 h SinV infection, Neuro 2a cells had a lower respiratory reserve capacity [34]. The Leak flux control ratio (L/E), which reflects the extent of intrinsic uncoupling, did not show any difference between samples, where the average ratios were 0.22 and 0.26 for mock-infected and infected cells, respectively. The fraction of oxygen consumption rate utilized for ATP synthesis, represented by the netR/E, significantly increased from 0.30 in mock-infected to 0.40 in Neuro 2a cells after 15 h of SinV infection. These results indicate that 30% (mock-infected) and 40% (SinV-infected) of ETS capacity were actually used for ATP synthesis, corresponding to an approximately 30% increase after 15 h of SinV infection. The slight decrease in Leak respiration in SinV-infected cells (Figure 4a) indicates an increase in oxygen consumption rate associated to F_1_F_o_ ATP synthase activity and, hence, coupled to ATP synthesis. Moreover, the decrease in ETS capacity (Figure 4a) contributed to the increase in respiration associated to ATP synthesis observed in infected Neuro 2a cells. Taking into account that cell viability is not affected after 15 h of infection (Figure 2b), the changes in mitochondrial respiratory functions appear to be early signs of cellular dysfunction during the course of SinV infection.

When the respiratory parameters of intact mock-infected and infected Neuro 2a cells were analyzed after 24 h of SinV infection, a different scenario was observed when compared to the time point of 15 h (Figures 4c and d). The largest difference was observed in ETS capacity, where SinV infection caused a significant 65% decrease in this parameter. The average rates for mock-infected and infected cells were 124.1 and 75.4 pmoles O_2_×10^6^ cells^−1^×seg^−1^, respectively. Routine respiration average rates were 67.2 and 54.8 pmoles O_2_×10^6^ cells^−1^×seg^−1^ and Leak respiration was 34.6 and 29.8 pmoles O_2_×10^6^ cells^−1^×seg^−1^ in mock-infected and SinV-infected cells, respectively. There were no significant differences between mock-infected and infected cells. The flux control ratios for the time of 24 h are shown in Figure 4d. The ratio R/E was even higher after 24 h of infection (0.56 *vs.* 0.76 in mock-infected and infected cells, respectively), indicating that in this situation infected cells presented an even less spare ETS capacity when compared to mock-infected cells. The L/E ratio was 0.28 for mock-infected and 0.40 for infected cells, respectively, and was significantly increased in infected cells, contrasting with the results observed in 15 h of infection and indicating that the apparent intrinsic proton leak is higher after 24 h of SinV infection when compared to mock-infected cells. The netR/E ratio was not different between groups and it was 0.28 and 0.36 for mock-infected and infected cells, respectively. This result indicates that, after 24 h, infected cells compensated for the partial uncoupling (increased L/E ratio) so that the same fraction of ETS was activated to drive ATP synthesis. An important observation is that flux control ratios of mock-infected cells in 15 h and 24 h were essentially the same, indicating that SinV infection affects cellular energy homeostasis and imposes its own metabolic program as suggested for human CMV infection [5].

In an attempt to further characterize the respiratory function and possibly unveil the mechanisms involved in SinV-induced mitochondrial bioenergetic alterations, oxygen consumption rates and respiratory parameters were measured in digitonin-permeabilized Neuro 2a cells.

**Figure 5:**
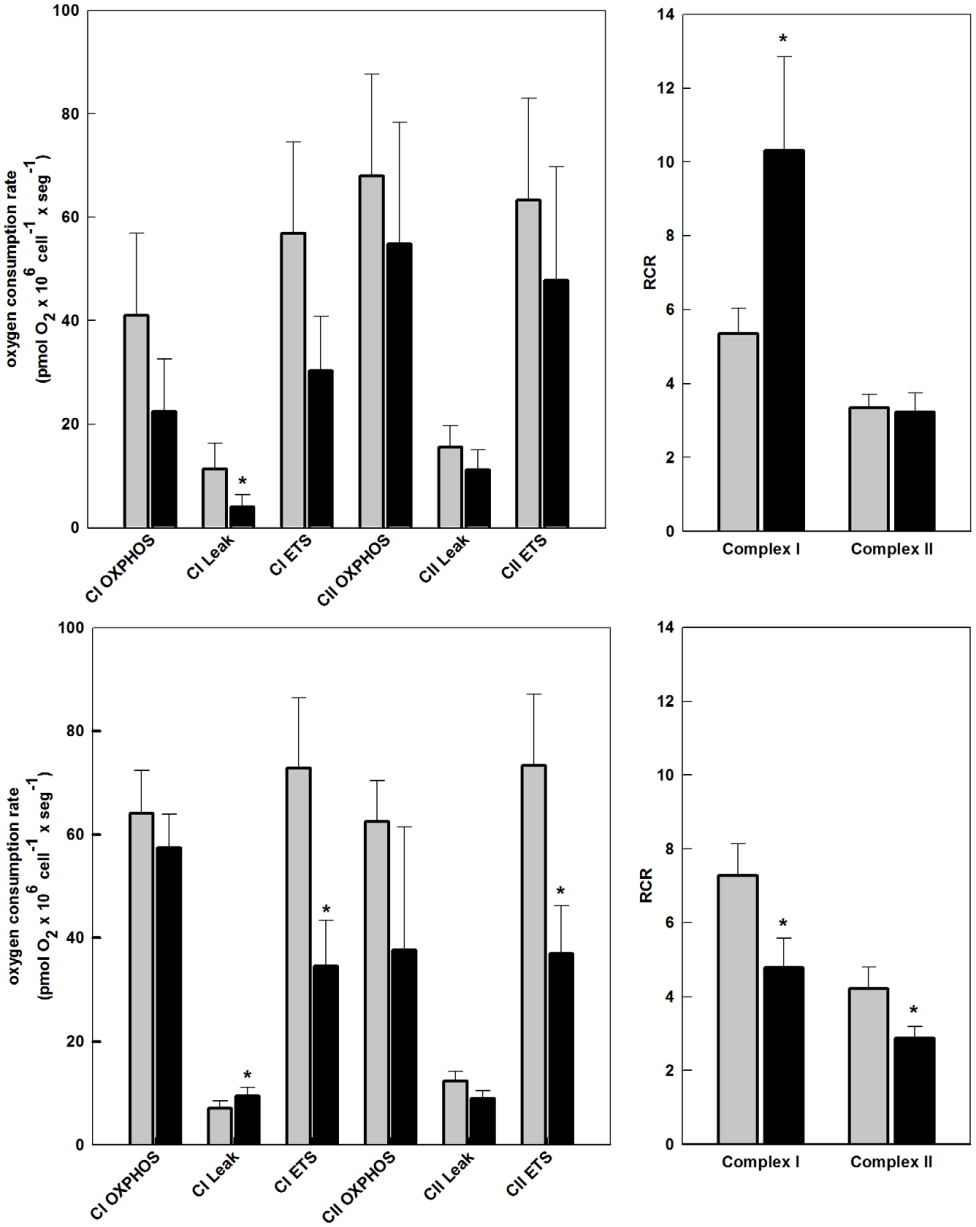
High-resolution respirometry reveals mitochondrial dysfunction in permeabilized Neuro 2a cells infected with SinV. Oxygen consumption rates were measured on digitonin-permeabilized Neuro 2a cells in mitochondrial medium MiR05. CI_OXPHOS_ oxygen consumption rates after addition of 5 mM pyruvate +5 mM malate +2.5 mM ADP; CI_Leak_ oxygen consumption rates after addition of olygomycin, supported by CI substrates; CI_ETS_ oxygen consumption rates after FCCP addition, driven by CI substrates; CII_OXPHOS_ oxygen consumption rates after addition of rotenone, 10 mM succinate +2.5 mM ADP; CII_Leak_ oxygen consumption rates after addition of olygomycin, supported by CII substrate; CII_ETS_ oxygen consumption rates after FCCP addition, driven by CII substrate. Respiratory control ratio (RCR) represents CI_ETS_/CI_Leak_ CII_ETS_/CII_Leak_ for complexes I and II, respectively. Mock-infected (gray bars) and SinV-infected (black bars) after 15 h (a,b) and 24 h (c,d). Values represent mean ± SEM; N = 10 for 24 h and N = 7 for 15 h, *P<0.05 compared to mock-infected cells.

The results presented in Figure 5a show that after 15 h of infection, there was a reduction, although not statistically significant, in oxidative phosphorylation capacity of Neuro 2a cells supported either by Complex I (CI; pyruvate/malate) or Complex II (CII; succinate) substrates. Additionally, maximum uncoupled respiration measured after FCCP addition did not differ in mock-infected and infected cells. Therefore, CI- and CII-dependent ETS capacity was not significantly affected after 15 h of SinV infection, contrasting to the results with intact cells, where a significant decrease in maximum uncoupled respiration was observed at this time point. On the other hand, Leak respiration supported by CI substrates, measured after oligomycin addition, presented a significant 77% decrease in SinV-infected cells (*P*<0.05). Leak respiration in intact Neuro 2a cells was also decreased after 15 h of SinV infection (Figure 4a).

It is well established that when cells are maintained in an appropriated medium containing reducing substrates that can donate hydrogen atoms (electrons and protons) to the respiratory chain, mitochondria start to respire at a slow rate due to the build up of a maximum proton gradient (state 2 or state 4 respiration) [35]. Once ADP and Pi are added, respiration is stimulated due to ADP conversion to ATP by F_1_F_o_-ATP-synthase using the proton gradient established across the inner membrane by the electron transport chain (state 3 respiration), and ΔΨ_m_ is partially reduced. When limiting amounts of ADP are added to mitochondria, state 3 respiration persists until ATP/ADP ratio approaches equilibrium, the proton gradient is increased again and oxygen consumption is reduced. In practice, any contamination of ATPase activity will prevent the restoration of a low respiration, i.e., state 4 respiration. For this reason, state 3 respiration can be terminated by addition of oligomycin, to inhibit ATP synthase, and to reach state 4-oligomycin respiration (state 4o respiration). If oligomycin addition is followed by FCCP addition, maximum uncoupled respiration is achieved (state 3 uncoupled). The parameter respiratory control ratio (RCR), defined as the ratio state 3 respiration/state 4 respiration seems to be the best general measure of mitochondrial function in permeabilized cells and isolated mitochondria [34]. Accordingly, RCR was calculated as state 3 uncoupled divided by state 4o for CI and CII substrates. Figure 5b shows that SinV-infected cells presented a two fold increase in CI RCR after 15 h, which was due to the significant decrease in state 4o respiration observed in SinV-infected cells, as shown in figure 5a. CI RCR for mock-infected and SinV-infected cells was 5.4 and 10.3, respectively. State 4o respiration is controlled mainly by the activity of proton leak [34]. Therefore, mitochondria of infected Neuro 2a cells seemed to maintain a sufficiently high *protonmotive force* to restrict electron transport, so that oxygen consumption rate not related to ATP synthesis is decreased after 15 h of SinV infection. This apparent improved function of mitochondria related to CI respiration probably adds up to the increased respiration related to phosphorylation observed in intact Neuro 2a cells (Figure 4b) and, likewise, seems to be early signs of bioenergetic alterations during the course of SinV infection. CII RCR was not different in mock-infected and SinV-infected cells (Figure 5b), suggesting a specific modulation of CI-related respiration.

As shown for intact Neuro 2a cells (Figures 4c and 4d), as infection progresses, mitochondrial dysfunction is easily seen in permeabilized Neuro 2a cells at the time point of 24 h (Figure 5c). The effects on respiratory parameters were more pronounced in the uncoupled state, where there was a significant decrease in ETS capacity linked to CI and CII respiratory complexes. The average values were 72.9 and 34.6 pmoles O_2_×10^6^ cells^−1^×seg^−1^, for CI ETS, and 73.3 and 37.0 pmoles O_2_×10^6^ cells^−1^×seg^−1^ for CII ETS, for mock-infected and SinV-infected cells, respectively. An interesting observation was that after 24 h of SinV infection, Leak respiration related to CI substrates was significantly increased, contrasting to the decrease in this parameter after 15 h of infection. The severe changes in both ETS capacity and in Leak respiration at 24 h of infection affected RCR as shown in Figure 5d. CI and CII RCR were significantly decreased after 24 h of SinV infection (*P*<0.05). RCR for CI was 7.3 and 4.8 and for CII was 4.2 and 2.9 for mock-infected and SinV-infected Neuro 2a cells, respectively.

It is of interest to mention that simultaneous addition of CI and CII substrates did stimulate oxidative phosphorylation capacity by 60% in both mock-infected and SinV-infected cells after 15 h (data not shown). On the other hand, after 24 h, no additive effect on respiration was seen in infected cells (data not shown). These results indicate that CII is also affected in this time point. The increase in respiration in these conditions is due to the convergent CI+II electron flow into the Q-junction as demonstrated [36].

SinV-replication efficiency was followed after each respirometry assay and viral titers were in the range of 5×10^7^ to 5×10^8^ pfu/mL (data not shown).

### SinV infection increases glucose uptake and glycolytic flux in Neuro 2a cells

**Figure 6:**
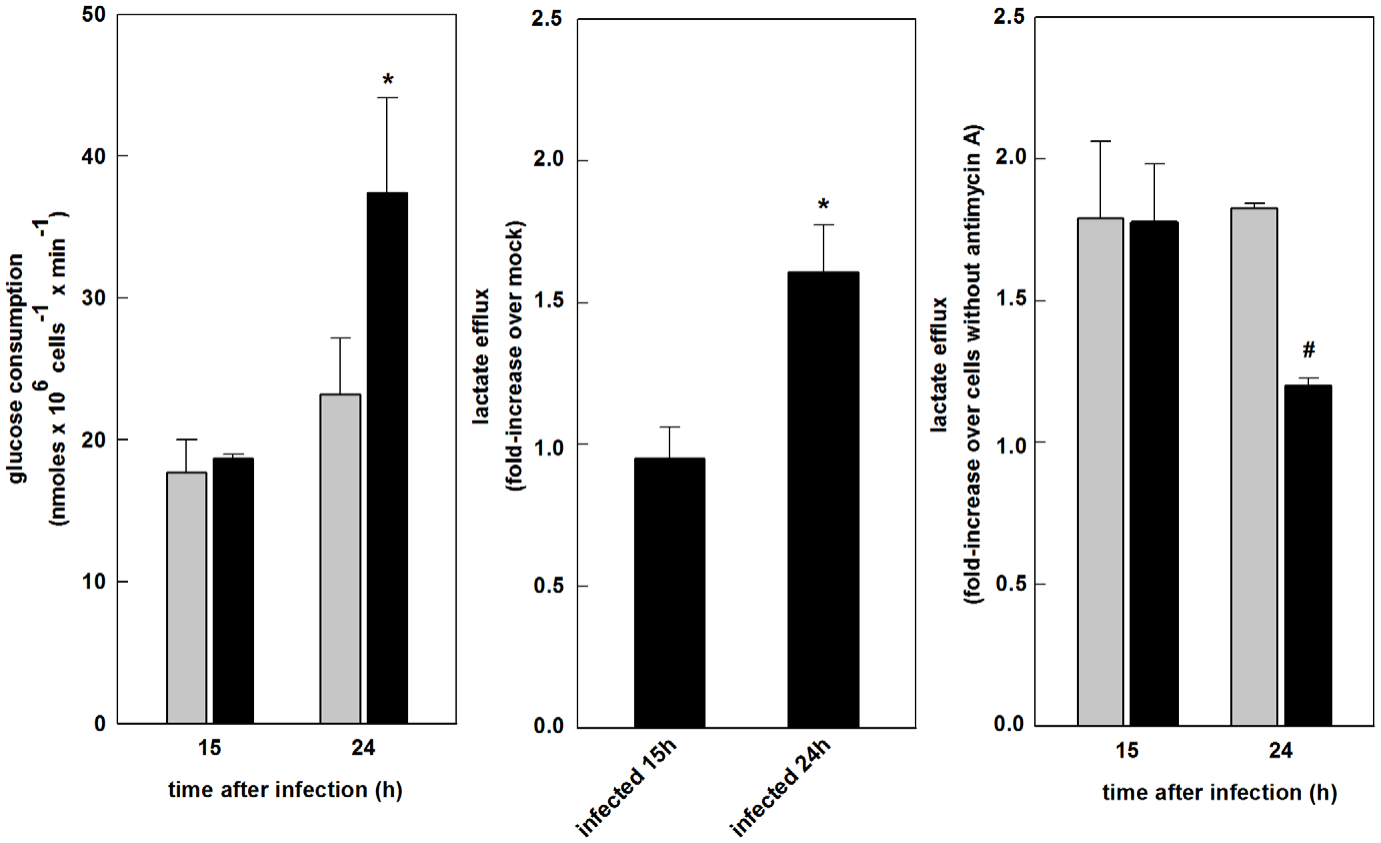
Effects of SinV infection on glucose uptake and glycolytic flux in Neuro 2a cells. Glucose uptake (a) and lactate efflux (b) were measured after 15 and 24 h of SinV infection. Antimycin effects on lactate efflux (c). Mock-infected (gray bars) and SinV-infected (black bars). Values represent mean ± SEM; N = 4, *P<0.05 compared to mock-infected cells; #*P*<0.05 compared to SinV-infected cells without Antimycin A.

It is well established that mitochondrial and glycolytic ATP production, in non-pathological situations, are reciprocally regulated so that ATP steady-state is maintained [37]. In order to search for possible associations between SinV-induced alterations in respiration and glucose metabolism, we analyzed glucose uptake and lactate efflux in Neuro 2a cells infected with SinV. Figures 6a and 6b show that the rate of glucose uptake and lactate efflux were not affected after 15 h of infection, whereas after 24 h, infected cells presented a significant 1.6 fold increase in both glucose consumption and glycolytic flux. Glucose consumption rates of mock-infected and SinV-infected cells were, respectively, 17.7 and 18.7 nmoles×10^6^ cells^−1^×min^−1^ after 15 h and 23.2 and 37.4 nmoles×10^6^ cells^−1^×min^−1^ after 24 h. There was a non-significant 30% increase in glucose consumption between 15 h and 24 h in mock-infected cells. On the other hand, the increase in glucose consumption in SinV-infected cells was significant at 24 h compared to 15 h after infection. Therefore, these results show that the degree of modulation of glucose metabolism follows mitochondria dysfunction: the more severe the dysfunction, the higher the flux through glycolysis.

The effects of antimycin A on glycolytic flux were also evaluated in Neuro 2a cells. Figure 6c shows that there is a significant 80% increase in lactate efflux in control cells treated with antimycin A, at 15 and 24 h. The same increment in lactate efflux was observed in infected cells after 15 h. On the other hand, after 24 h of infection, lactate efflux was stimulated only 20% after antimycin A addition. These results substantiate the findings that mitochondrial function is impaired in SinV-infected Neuro 2a cell.

### SinV infection affects cellular ATP content in Neuro 2a cells

**Figure 7:**
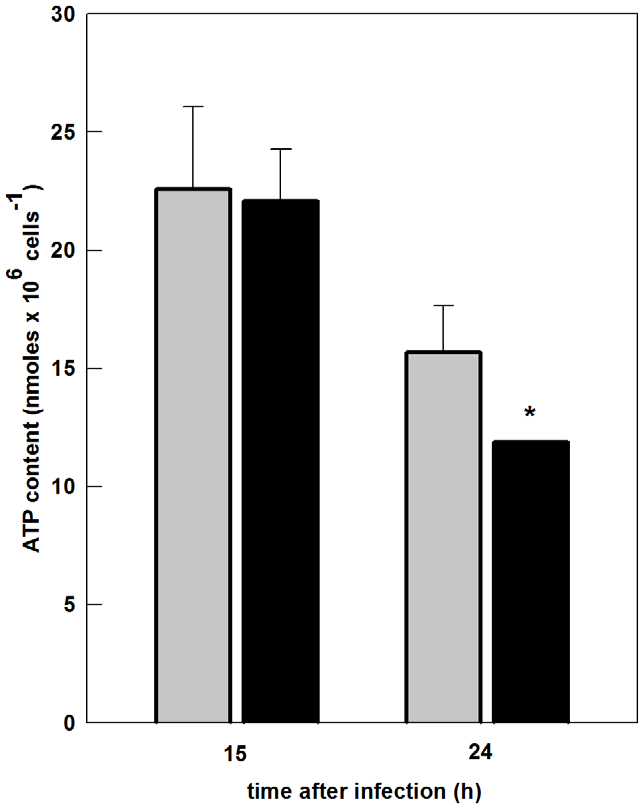
Effects of SinV infection on ATP content in Neuro 2a cells. ATP content was measured with HPLC after 15 and 24 h of SinV infection. Mock-infected (gray bars) and SinV-infected (black bars). Values represent mean ± SEM; N = 3, *P<0.05 compared to mock-infected cells; ^#^ *P*<0.05 compared to SinV-infected cells at 15 h.

Although the results showed in Figures 4b, 4d, 5a and 6 indicated that Neuro 2a cells infected with SinV did not display a decrease in respiration related to oxidative phosphorylation and also presented alterations in the flux through glycolysis, they did not rule out the possibility that SinV infection may have affected cellular ATP content. The results in Figure 7 demonstrated that after 15 h, the increase in netR/E ratio of infected cells, i.e, the fraction of ETS capacity activated to drive ATP synthesis, was sufficient to maintain ATP content in SinV-infected when compared to mock-infected cells. ATP content after 15 h was 22.6 for mock-infected and 22.1 nmoles ×10^6^ cell^−1^ for infected cells. Interestingly, after 24 h, despite the fact that mock-infected and infected cells utilized the same extent of ETS capacity (Figure 4d), and most importantly, that infected cells presented a significant increase in glycolytic flux (Figure 6b), ATP content was significantly decreased in infected cells when compared to non-infected ones. ATP content was 15.7 for mock-infected and 11.9 nmoles ATP ×10^6^ cell^−1^.

ATP content values were not significantly different from mock-infected Neuro 2a cells after 15 and 24 h. On the other hand, comparing the results from infected cells after 15 and 24 h, a significant 85% decrease was observed. These results indicate that mitochondrial dysfunction during the course of SinV infection may have compromised the energy homeostasis of Neuro 2a cells due to, at least in part, altered respiratory properties.

### SinV infection induces oxidative stress in Neuro 2a cells

**Figure 8:**
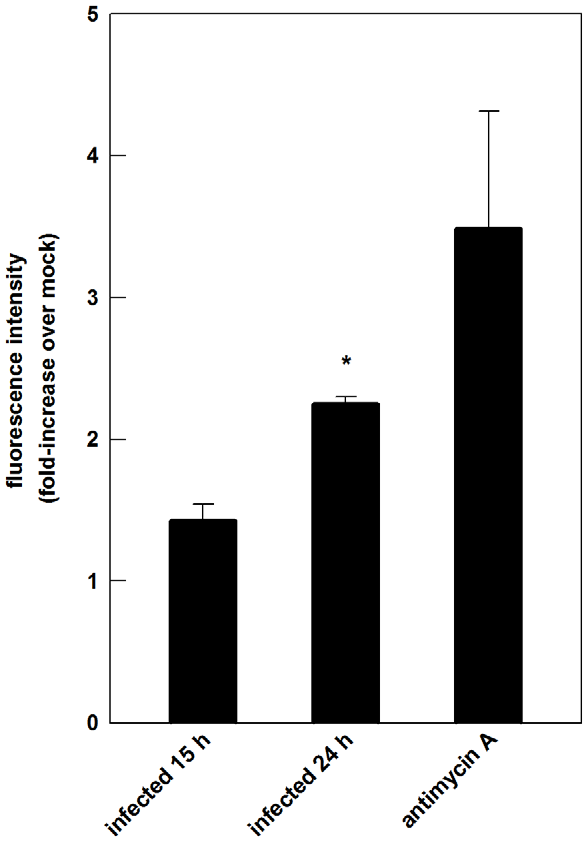
Reactive oxygen species accumulation after SinV infection in Neuro 2a cells. Reactive oxygen species were evaluated by following the oxidation of the probe CM-H_2_DCF after 15 and 24 h SinV infection. Antimycin A was used as a positive control. Values represent mean ± SEM relative to mock-infected cells; N = 3, *P<0.05 compared to mock-infected cells.

Reactive oxygen species (ROS) participate in many different ways in mitochondrial dysfunction. Accordingly, the effects of SinV on ROS accumulation were evaluated in Neuro 2a cells and are shown in Figure 8. At 15 h of infection, there was a non-significant 40% increase in ROS accumulation comparing to non-infected cells. On the other hand, ROS accumulation was significantly increased after 24 h SinV infection, suggesting that ROS might have contributed to mitochondrial dysfunction and SinV-induced Neuro 2a cells death. This corresponded to a 2.3 fold increase when compared to mock-infected Neuro 2a cells. ROS accumulation after Antimycin A addition on Neuro 2a cells is shown as a positive control.

## Discussion

Host cells provide the metabolic resources crucial for viral replication. Accordingly, the utilization of molecules rich in free energy for protein, membranes and viral RNA synthesis needs to be strictly controlled to ensure viral propagation. The mechanisms by which virus interact with host metabolism, altering and recruiting biosynthetic molecules for their own replication, on the other hand, remain unknown. Recently, it was proposed that viruses are metabolic engineers because of their ability to alter host energy metabolism to favor replication [1]. Although it has been well known for over 40 years that SinV replication modulates host cell metabolism by decreasing protein, RNA and phospholipids synthesis [16], [38], [39], [40], functional studies of mitochondria of infected cell and their relation to viral replication have not been reported. Several types of virus, which induce encephalitis in humans, such as the alphaviruses, frequently cause neurological damage in mice. In this respect, it was proposed that SinV is a good model for the investigation of virus-host interaction in neurons and its relationship with the progression of disease [16], [41]. In the present work, we showed that SinV infection of Neuro 2a cells, a mouse neuroblastoma cell line, presented characteristics similar to other well studied neuroblastoma cells, regarding the replication peak and apoptotic cell death. Here we show for the first time that mitochondrial bioenergetics is modulated during the course of SinV infection in such a way as to favor ATP synthesis required to support active viral replication.

Several reports have shown that an increased neuronal expression of Bcl-2 [24], [42], Beclin [25], Bax [26] and decreased expression of Apaf-1 on fibroblast [43] turned cells more resistant to apoptotic cell death induced by SinV infection. These results suggested that possible alterations on mitochondrial membrane permeability, which have profound impact on mitochondrial bioenergetics, might be related to SinV-induced cell death. According to the results presented in the present study, alterations on mitochondrial bioenergetics seem to be early events of SinV infection on Neuro 2a cells since ETS capacity was significant altered irrespective of effects on cell viability (Figures 4a and 5a). We have previously shown that alterations in mitochondrial bioenergetics seemed to play an important role on Dengue virus infection of human hepatic cells, and that these alterations also preceded cell death [17]. We reasoned that the decrease in ETS capacity might be related to a decrease in the activity of the respiratory complexes and/or alterations on substrate uptake and metabolism [34]. Since the decrease in ETS capacity was aggravated after 24 h of infection, reflected by the increase in the ratio R/E (Figure 4d), a decrease in ETS complex activity suggested itself. Indeed, after 24 h of infection, the results with permeabilized cells indicate that mitochondria of SinV-infected cells present a decreased capacity for substrate oxidation, given that ETS capacity for both CI and CII substrates is significantly decreased, as well as the RCR related to CI and CII (Figures 5c and 5d).

Although this is the first time that alterations on respiratory complexes are investigated in SinV infection, it was shown recently that patients infected with hepatitis C virus and HIV presented alterations on liver mitochondrial respiratory functions due to a decrease in Complex IV (CIV) activity [44]. Since CI- and CII-dependent ETS capacity deteriorated as SinV infection progresses and was severely compromised at 24 h, a defect on CIV might be suggested [34]. These observations together suggest that different virus utilize the same strategies to modulate host metabolism to favor and direct replication.

Since there were no differences in Routine respiration between SinV-infected and mock-infected cells, the decrease in ETS capacity at 15 h resulted in an increased in R/E ratio. Therefore, Routine respiration of infected Neuro 2a cells corresponded to an increased fraction of ETS capacity, which indicates that cells are working near their bioenergetic limit [34], and reflected a metabolic situation of an increased demand for ATP [37], [45]. In addition, the increase in the netR/E (Figure 4b) indicates that infected cells use a higher fraction of ETS capacity to drive ATP synthesis. Given that at 15 h viral replication peaked and cells were viable, these alterations on mitochondrial bioenergetic parameters appear to be a viral mechanism to support replication, since, as mentioned, this process is costly in terms of energy. Indeed, cell energy homeostasis was not affected by SinV infection, as ATP content at 15 h was similar in both mock and infected cells (Figure 7). The observation that CI RCR is increased in infected cells due to a decrease activity of proton leak (Figure 5a) possibly played an important role in the increase in netR/E ratio and might represent a compensatory mechanism for the decrease in ETS capacity. Surprisingly, glucose uptake and lactate efflux were not affect in Neuro 2a cells after 15 h of infection. Therefore, the apparent improvement in mitochondrial function seemed to play a major role in the maintenance of steady-state concentration of ATP. A decrease in ATP utilization by cellular processes possibly also contributed to energy homeostasis of Neuro 2a cells.

As infection progresses, cell viability is severely decreased and both apoptotic and necrotic cell death are detected (Figure 3), and respiratory functions seemed to deteriorate. After 24 h of SinV infection, Neuro 2a cells presented even less spare respiratory capacity, indicated by the 0.76 R/E ratio (Figure 4d). It has been proposed that neuronal dysfunction can be measured by its ability to respond to an increase in ATP demand [46]. Therefore, despite the fact that infected cells utilized the same proportion of ETS capacity to drive ATP synthesis as mock-infected cells – which indicates a constant rate of oxidative phosphorylation between samples – SinV infection promoted a decrease in total ATP content after 24 h (Figure 7). Additionally, mitochondrial modulation was followed by a significant increase in both the uptake and utilization of glucose molecules through the glycolytic pathway. Even so, ATP content was decreased. Therefore, the inability of Neuro 2a to increase ATP production to cover for the energy cost of viral replication and other cellular processes culminated in an energy collapse of Neuro 2a cells.

It has already been reported that the modulation of glucose metabolism is a characteristic of the infection by distinct alphaviruses, including SinV [2] and Mayaro virus [3]. Additionally, it was shown that cells infected with different constructs of Hepatitis C virus showed a marked mitochondrial dysfunction due to viral protein synthesis, which was compensated by an increase in glycolytic flux mediated by the transcription factor HIF-1α [47]. In the present study, despite the fact that the increased glucose utilization did not compensate for ATP content, this fact could also be important for the production of biosynthetic precursors via an increased flux through the pentose phosphate pathway (PPP). A recent work suggested that an increased flux through glycolysis and deviation of glucose carbons to the PPP is a strategy utilized by viruses with long replication cycle [48]. It remains to be determined its relation to the replication of SinV in Neuro 2a cells.

Alterations in ATP content seem to be a common event in alphavirus infection. Indeed, we showed that Mayaro virus infection also decreased ATP content of Vero cells, which was related to virus-induced cell death [3]. Neurons may display, simultaneously, different types of death and in these cases mitochondria play a central role [49]. In this respect, the modulation of mitochondrial bioenergetics might have affected the type of cell death following SinV infection of Neuro 2a cells. It can be seen in Figure 3 that apoptosis overcame necrosis after 24 h of SinV infection. The increase in netR/E ratio possibly accounted for the increased CI RCR, which contributed to energy homeostasis, might have supported apoptosis, since ATP is required to sustain and to induce the formation of apoptosome [50]. As infection progressed and mitochondrial dysfunction worsened, ATP content decreased leading to necrosis. It has already been shown that apoptotic cell death plays an important role on neuronal dysfunction during SinV infection [16]. As a result, it is important to stress out the strict association between the early modulation of mitochondrial bioenergetics by SinV and the outcome of infection and Neuro 2a cells dysfunction.

Cellular responses to oxidative stress have also been shown to modulate the outcome of SinV infection, where increased antioxidant activity resulted in increased cell viability and persistent neuronal infection [27]. It is widely recognized that mitochondrial oxidative metabolism is an important source of ROS in eukaryotic cells. Additionally, oxidative stress plays a central role in the development and of many if not all neurodegenerative diseases [51]. The fact that ROS accumulation is not altered after 15 h of infection and significantly increased after 24 h suggests that oxidative stress contributed to mitochondrial dysfunction and to Neuro 2a cells death. An important observation is that Leak respiration tended to be decreased in intact and permeabilized Neuro 2a-infected cells after 15 h (Figures 4a and 5a). A decrease in Leak respiration corresponds to an increased coupling of oxygen consumption and ATP synthesis, and this has been implicated in increased mitochondrial ROS production due to an increase in ΔΨ_M_ [52]. We could speculate, therefore, that the cumulative effect of decreased Leak respiration at 15 h is the increase in ROS accumulation observed after 24 h of SinV infection. Lower mitochondrial respiratory rates, caused by decreased respiratory complexes activities, have also been implicated in increased mitochondrial ROS release [53], [54], [55]. Consequently, the results presented in Figures 4 and 5, showing the decrease in ETS capacity after SinV infection, might also explain the increased ROS accumulation after 24 h. In this respect, SinV infection affected mitochondrial bioenergetics, which might have contributed to an increased production of ROS. This, in turn, aggravated mitochondrial function resulting in Neuro 2a cell death. The increase in the L/E ratio after 24 h of SinV infection (Figure 4d) and the increase in CI Leak respiration (Figure 5c), indicating altered proton leakiness, corroborate this fact since it indicates that infection promote an increase in mitochondrial inner membrane permeability and ΔΨ_M_ collapse.

One possible link between mitochondrial dysfunction and oxidative stress during SinV infection of Neuro 2a cells is calcium metabolism. It has been shown that mitochondrial calcium overload is associated to mitochondrial oxidative damage [49], [51] and calcium imbalance has also been implicated in neurodegeneration [56]. It has been demonstrated that SinV infection induces an increase in calcium influx [57], and that infection of Semliki Forest virus, which is also an alphavirus, resulted in an increase in mitochondrial calcium uptake [58]. These data favor the hypothesis that alterations on calcium homeostasis play a role on mitochondrial dysfunction of Neuro 2a cells infected with SinV.

In this work, we demonstrated for the first time that SinV infection affects the respiratory function of mouse neuroblastoma cells Neuro 2a. According to our results, the modulation of mitochondrial bioenergetics significantly affected cellular ATP content and this was synchronous with SinV replication cycle and cell death. These alterations in mitochondrial bioenergetics appear to be early cellular responses and, therefore, might represent cellular determinants of the outcome of SinV infection.

Recent studies showed that respiratory complexes activity of lymphocytes from patients [59] and of human hepatic cells [60] infected with HIV were altered upon retroviral therapy, pointing out to the fact that functional studies of mitochondria might be helpful to chose adequate anti-viral therapy and as a means to follow treatment. Whilst further studies are necessary to disclose the molecular mechanisms underlying SinV-induced encephalitis and neuronal death, the groundwork established in the present work suggests that cell respirometry analysis for the investigation of mitochondrial bioenergetics of neuroblastoma, especially at the beginning of infection, may be an important tool for understanding SinV-host cells interactions.

## Materials and Methods

### Cell culture and virus propagation

Neuro 2a, a mouse neuroblastoma cell line, was purchased from the Rio de Janeiro Cell Bank (Rio de Janeiro, RJ, Brazil). The cells were grown in Dulbecco's modified Eagle's medium (DMEM - Invitrogen Life Technologies, CA, USA), 5 mM glucose, pH 7.4, supplemented with 10% fetal bovine serum (FBS; Invitrogen Life Technologies, CA, USA), at 37°C, in humidified incubation chamber with 5% CO_2_. Cells were seeded at a density of 1×10^6^ cells per plastic petri dish (100 mm diameter). Cells were grown for 2 days until they reached 80% confluence and were either mock-infected or infected with SinV strain AR339. Virus stocks were prepared in BHK-21 cells grown in α-Minimum Essencial Medium (α-MEM Invitrogen Life Technologies, CA, USA) supplemented with 10% FBS. After 2 days of propagation, cell debris were removed by centrifugation at 1,000× g for 5 min, and the supernatant was stored at −80°C. Virus stocks titers were determined by plaque assay, ranging from 10^7^ to 10^8^ PFU/mL.

### Virus quantification

#### Plaque Assay

SinV infectious particles released from infected cells were quantified by determining virus titers in the culture medium by plaque assay in BHK-21 cells. Cells were seeded in 12-well dishes in α-MEM supplemented with 10% FBS in a humidified incubator at 37°C in 5% CO_2_ for 1 day until they reached confluence. Medium was removed and the monolayer was infected with 200 µl of serially diluted culture medium collected from infected cells. The dishes were incubated for 1 hour at 37°C for viral adsorption. Subsequently, 2.0 ml of 1% carboximetilcelulose (CMC; Sigma-Aldrich, St. Louis, MO, USA) mixed with α-MEM supplemented with 5% FBS were added to each well. The dishes were maintained in a humidified incubator at 37°C, 5% CO_2_ for 2 days when cells were fixed with 10% formaldehyde at room temperature and the CMC plugs removed by washing with water. Plaques were visualized by staining with 1% crystal violet in 20% ethanol solution.

#### Flow cytometry

Infected cells were also quantified by flow cytometry. After 6, 15 and 24 h of infection, cells were washed with phosphate buffer, harvested, and fixed in 4% paraformaldehyde for 15 min. Subsequently, cells were treated with 0,1% saponin in PBS and then incubated with blocking solution (phosphate buffer supplemented with 2% FBS and 0.1% bovine serum albumin) for 30 min, at room temperature. Then, cells were incubated for 1 h with mouse anti-Eastern Equine Encephalitis virus monoclonal antibody (Chemicon International, Millipore), an antibody that reacts with an E1 epitope shared by all alphaviruses, washed and stained with anti-mouse IgG conjugated to Alexa fluor-488 (Invitrogen, Carlsbad, CA) for 30 min. The percentage of SinV-infected cells was evaluated by using a FACScalibur cytometer (Becton Dickson Immunocytometry System). For each sample, 10,000 events were acquired and analyzed using the CellQuest software.

### Flow cytometry for characterization of cell death

Apoptosis/necrosis was measured using a double staining method with The Vybrant Apoptosis Assay Kit#2 (Molecular Probes). Neuro 2a cells were infected with SinV MOI 5 for 15 and 24 h. After the infection period, cells were processed according to manufacturer's instructions. 400 µL of 1× annexin-binding buffer were added and stained cells were analyzes in FACSCalibur flow cytometer using CellQuest software, respectively, for data acquisition and analyses, at 530 nm fluorescence emission.

### Cell morphology and viability

Morphological changes of Neuro 2a cells infected with SinV after 15 and 24 h were observed under a phase-contrast Olympus IX51 microscope. Approximately 10 fields were analyzed in each condition and photographs were taken at a 200× magnification. Cell viability was determined at 6, 15 and 24 h after infection using Trypan Blue exclusion and 3-(4,5-dimethylthiazol-2-yl)-2,5-diphenyl tetrazolium bromide (MTT) (USB Corporation, Ohio, USA) assays.

### Oxygen consumption analysis and calculation of respiratory parameters

Mitochondrial oxygen consumption rates were monitored and evaluated by high-resolution respirometry with Oxygraph-2 k (Oroboros Instruments, Austria). This instrument provides sufficient sensitivity to detect subtle changes in cellular respiration and allows the utilization of small sample size [61]. Oxygen consumption rates were measured in intact Neuro 2a cells, suspended in the culture medium (DMEM, 5 mM glucose) without fetal bovine serum, at cell density of 2.5×10^6^ cell per mL, at 37°C in 2 mL chamber, at stirring rate of 750 rpm, as described elsewhere [62]. Briefly, after each time of infection, cells were harvested with trypsin and washed twice with DMEM supplemented with serum for protease inactivation. Cells were counted and viability checked with trypan blue dye. In each experiment, oxygen consumption rates were determined in a time interval up to 1 hour. At the end of each experiment, cell viability was evaluated and it was found to be similar between mock-infected and SinV-infected cells. After cells were added to the respiration chamber, Routine respiration (R) was measured in the coupled state. Subsequently, 3 µg/mL oligomycin was added to record non-coupled respiration or Leak respiration (L). Oxygen consumption in the presence of oligomycin represents the sum of proton leak through the inner mitochondrial membrane plus any non-mitochondrial oxygen consumption. Respiration was completely blocked after the addition of antimycin A, which reveals that the non-mitochondrial oxygen consumption in Neuro 2a cells was negligible (data not shown). Oligomycin inhibits mitochondrial phosphorylation system and Leak respiration corresponded to oxygen consumption uncoupled to ATP synthesis. The maximum uncoupled respiration was measured in the presence of optimum carbonyl cyanide p-(trifluoromethoxy) phenylhydrazone (FCCP) concentration (200 nM). Optimum FCCP concentration was determined after FCCP titration (40–500 nM, data not shown).

Maximal uncoupled respiratory activity is a measure of Electron Transport System (ETS) capacity (E). Since this was recorded on intact cells, it reflects ETS capacity under physiological substrate supply. ETS capacity was used to normalize and calculate respiratory flux control ratios. Routine flux control ratio (R/E) reflects mitochondrial activity related to maximal ETS capacity and corresponds to how much spare respiratory capacity the cells posses [34]. Leak flux control ratio (L/E) reflects Leak respiration as a function of ETS and corresponds to the extent of intrinsic uncoupling. Finally, net Routine flux control ratio, netR/E, which is calculated by (R-L)/E, is the fraction of ETS capacity used to drive ATP synthesis [38].

For respiratory complexes activities and respiratory control ratio (RCR) calculations, permeabilized Neuro 2a cells were used. Cells were suspended in mitochondrial respiration medium Mir05 [63]. Immediately after the addition of cells (approximately 5×10^6^ cells), digitonin (0.005%, w/v) was added, followed by respiratory inhibitors and substrates. Oxidative phosphorylation capacity driven by Complex I (CI) was determined by the addition of 5 mM pyruvate and 5 mM malate followed by the addition of 2.5 mM ADP. Complex II (CII) oxidative phosphorylation capacity was measured after the addition of 1 µM rotenone (CI inhibitor) and 10 mM succinate, followed by 2.5 mM ADP. Leak respiration was accessed after recording CI and CII oxidative phosphorylation capacity by the addition of 3 µg/mL oligomycin. Maximum uncoupled respiration was measured after addition of 200 nM FCCP.

As in the respirometric analysis with intact cells, maximum uncoupled respiration in permeabilized cells is a measure of ETS capacity. Since in this case CI and CII substrates were added separately, ETS capacity was accessed individually for each complex. RCR represented the oxygen consumption rates in CI or CII uncoupled states divided by the leak oxygen consumption rates and was calculated for each respiratory complex substrate. Oxidative phosphorylation capacity was also measured by convergent CI+CII electron flow into the Q-junction [36]. In this case, after addition of CI substrates and ADP, succinate was added (data not shown).

Data acquisition and analysis were done with DatLab 4.3 software (Oroboros Instruments, Innsbruck, Austria).

### Glucose uptake and lactate efflux to the culture medium

Glucose uptake and lactate efflux was evaluated by Nuclear Magnetic Resonance (NMR). After 15 h and 24 h, culture medium was replaced with fresh DMEM (no glucose) supplemented with 5 mM D-[U-^13^C]glucose. At a 15 min intervals (0–180 min), 500 µl aliquots from culture medium were collected to evaluate glucose disappearance from and lactate efflux to the culture. The same volume of medium was replaced to achieve a constant D-[U-^13^C]glucose concentration during the experiment. Antimycin A (2 µg/mL) was added to cells to evaluate the effects of mitochondrial respiration inhibition on the increment of glycolytic flux and to possibly detect mitochondrial dysfunction in SinV-infected Neuro 2a cells. Antimycin A effects were evaluated during a 180 min interval after 15 and 24 h of SinV infection. After this period, cell viability, accessed by trypan blue dye exclusion assay, was not affected.

One-dimensional ^13^C spectra for the kinetics of glucose uptake and lactate efflux were obtained at 28°C using 45° pulses with a repetition time of 0.6 s, 16000 complex points, 2028 scans and a spectral width of 200 p.p.m. The free-induction decays were zero-filled to 16384 points and apodized with exponential multiplication using line broadening of 10 Hz. Spectra were acquired with a Bruker DRX 400 MHz using a triple resonance probe (TXI). Spectral processing and analysis was performed using Topspin 2.0. Analysis and assignment of glucose and lactate were obtained using the Human Metabolome Database v 1.0 [64]. Glucose concentration was determined by comparison with standard calibration curve (r^2^ = 0.9943). Lactate efflux was quantified according to peak height and expressed relative to mock-infected cells, for 15 and 24 h.

### Cellular ATP content

ATP of Neuro 2a cells was extracted as described in [17]. Briefly, protein was precipitated with cold trichloroacetic acid (6%, v/v). After 3 cycles of freeze/thaw, cellular extracts were centrifuged and kept at −80°C until analysis. ATP content was analyzed by reverse-phase ion-pair high-performance liquid chromatography (HPLC) in a LC-10AS chromatograph with a high-pressure phase mixer and a UV-visible detector (SPD-10A; Shimadzu, Japan) operated at 254 nm as described in [65]. Before injection, samples were neutralized with 2.5 M K_2_CO_3_. Samples and standard were eluted from the column (Hypersil ODS, Supelco Co.) as follows: Initial condition: Phase A 60% (10 mM tetrabutylammonium, 10 mM KH_2_PO_4_, 0.25% methanol) and phase B 40% (5.6 mM tetrabutylammonium, 50 mM KH_2_PO_4_, 30% methanol). Linear solvent gradient between phase A and phase B was achieved between 0 and 30 minutes, when solvent proportion was 40% and 60%, respectively. This proportion was kept until ATP elution. Identity of ATP peak was confirmed by spiking of samples with standard.

ATP was quantified by external calibration of peak areas of standard.

### Reactive oxygen species accumulation

Reactive oxygen species accumulation in Neuro 2a cells was measured 15 and 24 hours post-infection by the formation of the oxidized derivative of 5-(and 6-)-chloromethyl-2′,7′-dichlorodihydrofluorescein (CM-H_2_DCF, Molecular Probes). Cells seeded in 6-well culture dishes, washed with Balance Saline Solution and incubated with 2 µM CM-H_2_DCF for 30 min, at 37°C. Probe oxidation was monitored fluorometrically using a Labsystems type 374 plate-reader fluorometer. The excitation and emission wavelength were 485 and 538 nm, respectively. As positive control, antimycin A in a final concentration of 2 µg/mL was used.

### Statistical analysis

Statistical analyses were performed using Graphpad Prism 5.0 (Graphpad Software, Inc). Results are presented as means (±SE). Paired and unpaired Student's *t*-tests were used for comparison of mock-infected (control) and SinV-infected cells. Two-tailed *P* values<0.05 and *P* values<0.09 were considered statistically significant and statistical tendency, respectively.
